# Review of medical image recognition technologies to detect melanomas using neural networks

**DOI:** 10.1186/s12859-020-03615-1

**Published:** 2020-09-14

**Authors:** Mila Efimenko, Alexander Ignatev, Konstantin Koshechkin

**Affiliations:** 1 Digital Health Institute, Federal State Autonomous Educational Institution of Higher Education I.M. Sechenov First Moscow State Medical University of the Ministry of Health of the Russian Federation (Sechenov University), Moscow, Russia; 2 Moscow Scientific and Practical Center of Dermatology, Venereology and Cosmetology of Moscow City Health Department, Moscow, Russia; 3 Information Technology Department, Federal State Budgetary Institution Scientific Centre for Expert Evaluation of Medicinal Products of the Ministry of Health of the Russian Federation, Moscow, Russia

**Keywords:** Melanoma classification, Skin cancer, Deep learning neural network, Convolutional neural network, Fuzzy clustering algorithm

## Abstract

**Background:**

Melanoma is one of the most aggressive types of cancer that has become a world-class problem. According to the World Health Organization estimates, 132,000 cases of the disease and 66,000 deaths from malignant melanoma and other forms of skin cancer are reported annually worldwide (https://apps.who.int/gho/data/?theme=main) and those numbers continue to grow. In our opinion, due to the increasing incidence of the disease, it is necessary to find new, easy to use and sensitive methods for the early diagnosis of melanoma in a large number of people around the world. Over the last decade, neural networks show highly sensitive, specific, and accurate results.

**Objective:**

This study presents a review of PubMed papers including requests «melanoma neural network» and «melanoma neural network dermatoscopy». We review recent researches and discuss their opportunities acceptable in clinical practice.

**Methods:**

We searched the PubMed database for systematic reviews and original research papers on the requests «melanoma neural network» and «melanoma neural network dermatoscopy» published in English. Only papers that reported results, progress and outcomes are included in this review.

**Results:**

We found 11 papers that match our requests that observed convolutional and deep-learning neural networks combined with fuzzy clustering or World Cup Optimization algorithms in analyzing dermatoscopic images. All of them require an ABCD (asymmetry, border, color, and differential structures) algorithm and its derivates (in combination with ABCD algorithm or separately). Also, they require a large dataset of dermatoscopic images and optimized estimation parameters to provide high specificity, accuracy and sensitivity.

**Conclusions:**

According to the analyzed papers, neural networks show higher specificity, accuracy and sensitivity than dermatologists. Neural networks are able to evaluate features that might be unavailable to the naked human eye. Despite that, we need more datasets to confirm those statements. Nowadays machine learning becomes a helpful tool in early diagnosing skin diseases, especially melanoma.

## Introduction

Melanoma is one of the most malignant and rapidly progressing neoplasms registered in humans. The American Cancer Society estimated that in 2019, about 96,480 new cases of melanoma would be detected in the United States, and the death rate will be 7230 [1]. The statistics of the Ministry of Health of the Russian Federation showed the incidence rate of melanoma in 2015 for 6.99 per 100 thousand people: the incidence among women is higher (7.97) than among men (5.86) [2].

## Background

Despite the great significance of the problem, there is still no substantial etiological factor for melanoma. Established that the risk of malignant melanocytes is increased by the ultraviolet radiation of a certain wavelength of type A and B (290–400 nm) [3]. On the one hand, it is established that researchers detected rare mutations, often inherited, which significantly increased the risk of melanoma. One class of mutations affects the gene CDKN2A (cyclin-dependent kinase inhibitor 2A) [4], which leads to the destabilization of p53, a transcription factor involved in apoptosis. Loss of the function of CDK4 (cyclin-dependent kinase 4) [5], a kinase involved in cell differentiation, is also associated with this gene. It is proved that people with mutations in the MCR1 gene responsible for red hair color have a risk of developing melanoma twice as high as the rest of the population [6, 7]. Familial cases of melanoma (FAMMM) are also associated with CDKN2A mutations, but this factor has low specificity.

Habitat plays a role: families living in areas with a high degree of insolation have a greater risk of melanoma occurrence; especially if all family members have light skin color (people with fair skin have a 20-fold higher risk of developing melanoma than representatives of the Negroid race) [7–9].

### Diagnosing melanoma

Visual inspection is the most common method for diagnosing skin melanoma. The most common is the ABCDE algorithm where A is «asymmetry», B is «borders», C is «color», D is «diameter», E is growing with time (evolving) [10, 11]. Some doctors prefer using E as an elevation above the skin. The combination of these signs has high sensitivity and specificity. A dermatoscopic method with such an algorithm allows detecting malignancy in early stages, especially in patients in risk groups for the development of melanoma. Patient education plays a significant role, including an explanation of the value and methods of periodic self-examination [11, 12].

### The usage or the application of neural networks in melanoma diagnosing

The problem of malignant skin diseases was relevant for people in any era. The first scientific literature on the treatment of melanoma appeared in the eighteenth century [13]. Rene Laennec first described melanoma as a disease [14]. General practitioner William Norris was the first to report on melanoma in English from Stourbridge, England, in 1820 [15]. The first official recognition of the progressive melanoma as non-treatable came from Samuel Cooper in 1840 [16]. He stated the only chance for a cure depends on the early treatment of the disease (that is, the early removal of a malignant mole).

In 1991, Cohen and Hudson published the work «Neural network approach to detection of metastatic melanoma from a chromatographic analysis of urine» [17]. The authors describe a neural net analysis of comparative data on urine chromatography of three groups of patients. Due to those findings, scientists noted great potential in the use of neural networks in the diagnosis of melanoma, having received the correct diagnosis from the neural network in more than 80% of cases. Afterward, neural networks for the diagnosis of melanoma were trained mainly for the analysis of dermatoscopic images. In 1994, scientists published a paper in which the neural network already conducts an analysis of color images. Their method was based on the shape and color of the tumor, which was analyzed by the neural network to classify the formations in images as malignant or benign. In their study, the authors received more than 80% of the correct diagnosis on real images of skin tumors [18].

### Objective

This study presents a review of PubMed papers including requests «melanoma neural network» and «melanoma neural network dermatoscopy». We review recent researches and discuss their opportunities acceptable in clinical practice.

## Methods

### Search strategy

For this review, we searched through the PubMed database. According to PubMed, the first publications for the request «melanoma neural network» date back to 1991. Since then, the number of publications has grown unevenly every year, and the peak was in 2005 (12 publications) and 2018 (22 publications). As of May 2019, there are 138 publications on this request. At the request «melanoma neural network dermatoscopy» PubMed provides a list of 40 publications most of which relate to 2016–2019 indicating a growing interest in the early diagnosis of melanoma by analyzing dermatoscopy images using machine learning. Only papers that reported results, progress and outcomes are included in this review. In total, we found 157 unique papers, and 11 papers were matching our criteria.

### Study selection

We have limited our review to articles that, in our opinion, are the most significant for assessing the diagnostic opportunities of neural networks. Only papers that showed results, progress and outcomes are included in this review. This latter criterion includes presenting the approaches in an understandable manner and discussing the results sufficiently as well as the process of neural network implementation resulted in overall progress.

## Results

### Neural networks nowadays

By 2019, the accuracy of diagnostics using the ABCD algorithm and its additions has increased and, according to some data, has begun to exceed the results of dermatologists. In a study published in March 2019, the neural network had a smaller percentage of erroneous results compared to dermatologists (145 people), which indicates higher reliability of computer vision compared to human assessment for the tasks of classifying dermatological images [19].

German scientists published a resonant study in 2016, which proposed a comparison to 58 dermatologists [20]. Discussion of this research in the scientific community has led many to conclude that convoluted neural networks can serve as an excellent diagnostic aid possessing greater sensitivity and specificity which will affect the cost of treatment, in particular, reducing the number of unnecessary operations. Regardless of the experience of any physician, we can benefit from helping to classify images with a neural network.

At a certain point, researchers faced the problem of image quality and the amount of noise on them. Researchers using FC (fuzzy clustering) models took up this. Clustering was included in the study with a convoluted neural network, which significantly increased the quality of analysis of dermatoscopic images.

In a recent study, researchers suggested that errors in diagnosing malignancy of melanomas could be associated with damage to images and the criterion of the boundaries of melanoma [21]. They decided to include in the backpropagation (BP) neural network an algorithm to improve the image quality and modify the boundary criterion, ultimately having the result identical in specificity but exceeding other methods in sensitivity and accuracy. They concluded that previously proposed characteristics of the boundaries of the formations were too sensitive to noise in the images, which led to a decrease in the sensitivity and accuracy.

Scientists studying FC models in convolutional neural networks (CNN) in their study concluded that using an effective training mechanism, a neural network can detect signs of various skin diseases of the same patient, as well as various diseases of numerous patients. This makes it possible to use neural networks in the diagnosis of a wider range of skin diseases [22].

In 2017, Nature published a study in which researchers trained neural networks to distinguish keratinocyte carcinomas from benign seborrheic keratosis and malignant melanomas from benign nevi. They said that the fast, scalable method could be on mobile devices and had the potential for significant clinical impact, including it in primary care and improving clinical decision-making for specialists in dermatology [23].

In 2018, a hybrid of the World Cup Optimization (WCO) algorithm and an artificial neural network was proposed, which made it possible to avoid falling into a local minimum and increased the speed of analysis. This approach has expanded the capabilities of the neural network and increased the frequency of making correct diagnoses [24]. In the same year, a multiparameter neural network algorithm was proposed. At the input level, this algorithm included patient history data such as gender, age, disease history (bronchial asthma, diabetes mellitus, cardiovascular diseases), ethnicity, etc. This algorithm is cheap, non-invasive, easy to use, and could become available for patients to calculate the risk without the direct participation of the doctor. The researchers plan to expand the list of factors, including the cases of ultraviolet irradiation [25]. In our opinion, the inclusion of additional parameters, such as data from genetic research and family history, can significantly improve the output of the neural network.

All those studies are based on the fact that the most important factor for a good prognosis for melanoma is early diagnosis. In 2018 Indian researchers trained the neural network to analyze images from “handheld imaging devices” instead of from stationary dermatoscopic devices [26]. Such more accessible devices give more prospects for early correct diagnosis. In general, we can conclude that for the macro images, the proposed model provides optimal efficiency in the diagnosis of melanoma.

In addition, German researchers performed a new study in 2019. Results showed that a convolutional neural network trained by open-source images outperformed 136 of the 157 dermatologists and all the different levels of experience [27].

## Discussion

The analysis of this study leads us to the following statements:

- Neural networks in 2019 have greater sensitivity and specificity than dermatologists
- A neural network can evaluate features that might be unavailable to the naked human eye
- CNN provides the possibility of early detection of signs of melanoma and early treatment
- The need for staff training and the purchase of expensive equipment for creating dermatoscopic images can be replaced by software using FC-neural networks

By 2019, neural networks show significantly higher sensitivity, specificity and accuracy in comparison with the previous versions and work of dermatologists. The prospect of using this method as an auxiliary and later, perhaps, the main diagnostic is expressed in its increasing availability, accuracy and ease of use.

## Conclusions

In the age of rapid technology development, information systems and machine learning, the neural network is a learning algorithm that can greatly facilitate and improve the level of accuracy and timeliness of diagnostics.

## Data Availability

All papers are available on publisher websites. All data generated or analyzed during this study are included in this published article.

## Abbreviations

BP: Backpropagation
CNN: Convolutional neural networks
FAMMM: Familial Atypical Multiple Mole Melanoma Syndrome
FC: Fuzzy clustering
WCO: World Cup Optimization
ABCD algorithm: Algorithm based on asymmetry, border, color, and differential structures
